# Multilayer Membranes of Glycosaminoglycans and Collagen I Biomaterials Modulate the Function and Microvesicle Release of Endothelial Progenitor Cells

**DOI:** 10.1155/2016/4796578

**Published:** 2016-04-13

**Authors:** Bingyan Dai, Qunwen Pan, Zhanghua Li, Mingyan Zhao, Xiaorong Liao, Keng Wu, Xiaotang Ma

**Affiliations:** ^1^Guangdong Key Laboratory of Age-Related Cardiac and Cerebral Diseases, Affiliated Hospital of Guangdong Medical University, Zhanjiang 524001, China; ^2^Department of Orthopaedics, Wuhan Third Hospital, Wuhan, Hubei 430000, China; ^3^Department of Stem Cell Research Center, Affiliated Hospital of Guangdong Medical University, Zhanjiang 524001, China; ^4^Department of Cardiology, Affiliated Hospital of Guangdong Medical University, Zhanjiang 524001, China

## Abstract

Multilayer composite membrane of biomaterials can increase the function of adipose stem cells or osteoprogenitor cells. Recent evidence indicates endothelial progenitor cells (EPCs) and EPCs released microvesicles (MVs) play important roles in angiogenesis and vascular repair. Here, we investigated the effects of biomaterial multilayer membranes of hyaluronic acid (HA) or chondroitin sulfate (CS) and Collagen I (Col I) on the functions and MVs release of EPCs. Layer-by-layer (LBL) technology was applied to construct the multilayer composite membranes. Four types of the membranes constructed by adsorbing either HA or CS and Col I alternatively with different top layers were studied. The results showed that all four types of multilayer composite membranes could promote EPCs proliferation and migration and inhibit cell senility, apoptosis, and the expression of activated caspase-3. Interestingly, these biomaterials increased the release and the miR-126 level of EPCs-MVs. Moreover, the CS-Col I membrane with CS on the top layer showed the most effects on promoting EPCs proliferation, EPCs-MV release, and miR-126 level in EPCs-MVs. In conclusion, HA/CS and Collagen I composed multilayer composite membranes can promote EPCs functions and release of miR-126 riched EPCs-MVs, which provides a novel strategy for tissue repair treatment.

## 1. Introduction

Medical biomaterials for the repair or replacement of tissue or organs are polymeric substances. Biomaterials like hyaluronic acid (HA), chondroitin sulfate (CS), and Collagen (Col) have biological activities on stem cells [1]. Fibril-forming Col I (Col I) as one of the most abundant matrix proteins can also affect the growth, spread, and differentiation of stem cells [2–4]. Glycosaminoglycans like HA and CS, as the extracellular matrix (ECM) components cooperating with numerous cell surface adhesive proteins (e.g., fibronectin) and cytokines (growth factors), can impinge on cell biological processes such as growth and differentiation [5, 6]. Moreover, CS can promote the organization of soluble Col I precursors into fibrillar form which is essential for ECM structure and function, affecting the activity of adipose stem cells [7].

Various surface modification techniques have been used to improve the bioactivity of biomaterials. The so-called layer-by-layer (LBL) technique has emerged as a simple and versatile method for physical surface modification. Macromolecules including Col I, HA, and CS have natural adsorption and adhesion characters which allow paving multilayer composite membrane. Col I and either CS or HA are considered to be suitable for making multilayer membranes that have ECM-like characteristics and functions [8, 9]. The LBL multilayer film with HA-Col I or CS-Col I as raw materials could modulate fibroblast cell and osteoprogenitor stem cell functions (proliferation, migration, and regeneration) and might be used for making the bioactive coatings of implants [1, 10]. However, whether the components (HA-Col I or CS-Col I) and top layer (HA, CS, or Col I) of multilayer biomaterial membranes affect EPC activities remains unclear.

Endothelial progenitor cells (EPCs) are precursors of endothelial cells mobilized from bone marrow to peripheral blood in response to ischemia or injury [11, 12]. In recent years, many groups have reported the therapeutic effects of EPCs on tissue wound, heart infarction, and ischemic stroke [12–15]. It has been suggested that the therapeutic effects of EPCs are likely affected by pathological conditions of the body [16, 17]. Therefore, enhancing EPC function is a reasonable strategy to be applied for the treatment. Microvesicles (MVs) are lipid membrane vesicles with diameter of 0.1–1 *μ*m which are released when cells are activated or under apoptosis. They carry the characteristic of their mother cells and affect the function of recipient cells by transporting the contents of proteins, miRNAs (miRs), and mRNAs [18]. The therapeutic effects of EPCs are currently considered to be partly related to EPCs-MVs [19]. Moreover, it has been shown that miR-126 plays an important role in mediating EPCs-MV functions [20].

In this study, we investigated the effects of different multilayer membranes (HA-Col I or CS-Col I) with different top layers (HA, CS, or Col I) on EPC cell proliferation, migration, apoptosis, senescence, and MV release and miR-126 carry.

## 2. Materials and Methods

### 2.1. Preparation of Multilayer Composite Membranes

Multilayer membranes were fabricated on cleaned culture dish, respectively. Polyethyleneimine (PEI) was used at pH 7.4 as anchoring base layer to obtain a positive net charge of the respective substrates. Multilayers were formed on top of the PEI layer using CS or HA as polyanion and Col I as polycation. The polyanion layer (HA or CS) was adsorbed for 15 min at pH 4.0, while Col I layer was adsorbed for 20 min at the same pH value. Each adsorption step was followed by rinsing with 0.15 M sodium chloride solution (pH 4.0) for 3 × 5 min. As a result, biomaterial membrane systems with multilayers were obtained by adsorbing HA/CS and Col I alternatively on top of the PEI layer. We constructed four types of multilayer membranes. They were type 1 (defined as HA^*∗*^-Col I): three bilayers of HA-Col I with an additional layer of HA (seven alternative layers of HA and Col I with HA on the top), type 2 (defined as HA-Col I^*∗*^): four bilayers of HA-Col I (eight alternative layers of HA and Col I with Col I on the top), type 3 (defined as CS^*∗*^-Col I): three bilayers of CS-Col I with an additional seventh layer of CS (seven alternative layers of CS and Col I with CS on the top), and type 4 (defined as CS-Col I^*∗*^): four bilayers of CS-Col I (eight alternative layers of CS and Col I with Col I on the top). Untreated well plate was set as the vehicle group.

### 2.2. Culture of EPCs

Male adult (8–10 weeks of age; weight ranges from 20 to 25 g) C57/Bl mice were used for all experiments. EPCs were generated from bone marrow mononuclear cells (MNCs) as we previously reported [21, 22]. In brief, bone marrow was flushed out from tibias and femurs and MNCs were isolated by using density gradient centrifuge method. MNCs isolated from C57 mice were counted and plated on fibronectin-coated 6-well plates and then grown in endothelial cell basal medium-2 (EBM-2) supplemented with 5% FCS containing EPC growth cytokine cocktail (Lonza, Walkersville, MD, USA). After 3-day culture in wells not coated (vehicle group) or coated with biomaterial membranes, nonadherent cells were removed by washing with PBS. Thereafter, culture medium was changed every 2 days.

### 2.3. Cell Apoptosis Assay

Cell apoptosis was analyzed by Hoechst 33258 staining and Annexin V-PE/7-AAD apoptosis detection kit (BD Biosciences) as we previously described [23]. In brief, EPCs were seeded on culture wells not coated (vehicle group) or coated with biomaterial HA^*∗*^-Col I, HA-Col I^*∗*^, CS^*∗*^-Col I, or CS-Col I^*∗*^ membrane at a density of 2 × 10^5^/well in 2 mL serum-free EBM-2 medium. After culture for 24 h, cell apoptosis rates were measured. For Hoechst 33258 staining, cells were fixed and stained with Hoechst 33258 solution according to the manufacturer's instructions (Beyotime) followed by fluorescence microscope observation. Five independent fields were assessed for each well. The average number of positive cells and total cells per field (magnification, 200x) were determined. The apoptotic rate of cells was defined as the ratio of positive cells versus total cells. And for Annexin V-PE/7-AAD apoptosis detection, cells were washed with PBS, resuspended with 100 *μ*L 1x Annexin-binding buffer, incubated with 5 *μ*L PE-conjugated Annexin V and 5 *μ*L 7-aminoactinomycin (7-AAD) for 15 min in the dark, and then analyzed by flow cytometry. Cells stained with both Annexin V-PE and 7-AAD were considered to be late apoptotic EPCs, and those stained only with Annexin V-PE were considered to be early apoptotic cells. The experiment was repeated three times. And three plates per experiment were analyzed in each group.

### 2.4. EPCs Proliferation Assay

Proliferative capability of EPCs was tested by MTT (3-[4,5-dimethylthiazol-2-yl]-2,5-diphenyltetrazolium bromide) (Sigma, 5 mg/mL) assay. EPCs were seeded in 96-well plate not coated or coated with biomaterial membranes (HA^*∗*^-Col I, HA-Col I^*∗*^, CS^*∗*^-Col I, or CS-Col I^*∗*^) and cultured in 100 *μ*L EBM-2 (supplemented with 10% FBS) as described above. MTT solution (20 *μ*L) was added and incubated with cells for 4 h at 37°C; then 150 *μ*L DMSO was added to each well and incubated with the cells for 15 min at 37°C. The optical density (OD) value of cells was read at 490 nm in a microplate reader (BioTek). Cells in triplicate wells were examined at each time point, and the experiment was repeated for three times. Results were calculated from the values obtained in three independent experiments.

### 2.5. Migration Assay of EPCs

The migration distance of EPCs was measured by scratch assay. EPCs were grown to confluence on 6-well cell culture plate not coated (vehicle group) or coated with biomaterial membranes (HA^*∗*^-Col I, HA-Col I^*∗*^, CS^*∗*^-Col I, or CS-Col I^*∗*^). A scratch was made through the cell monolayer using a P200 pipette tip. After washing with PBS, cells were cultured in 0.5% FBS maintenance medium. Photographs of the wounded area were taken immediately (0 h) and 48 h after making the scratch to monitor the invasion of cells into the scratched area. Quantitative analysis of migration was measured as the following equation:

(1) $$\begin{array}{c} \frac{\left(\text{cell free area at }0\;h-\text{cell free area at }16\;h\right)}{\text{cell free area at }0\;h}\times 100\%. \end{array}$$

### 2.6. Cell Senescence Assay

Long-term culture was used to establish the senescent model of EPCs. In brief, EPCs were cultured in 6-well plate not coated (vehicle group) or coated with biomaterial membranes (HA^*∗*^-Col I, HA-Col I^*∗*^, CS^*∗*^-Col I, or CS-Col I^*∗*^) for 4 days. After that, *β*-Galactosidase Staining Kit (Beyotime, China) was used to analyze the senescent EPCs. In brief, after washing with PBS, EPCs grown on 6-well plate not coated (vehicle group) or coated with biomaterial membranes (HA^*∗*^-Col I, HA-Col I^*∗*^, CS^*∗*^-Col I, or CS-Col I^*∗*^) were treated with 2% formaldehyde and 0.2% glutaraldehyde in PBS for 6 min and then incubated with fresh X-gal staining solution (1 mg/mL X-gal, 5 mmol/L potassium ferrocyanide, 5 mmol/L potassium ferricyanide, and 2 mmol/L MgCl_2_; pH 6) for 12 h at 37°C without CO_2_. After staining, blue-stained cells and total cells were counted at 3 different microscopic fields. The percentage of *β*-galactosidase positive cells was calculated.

### 2.7. Preparation and Detection of EPCs-MVs

EPCs-MVs were generated from EPCs cultured as previously described [20]. In brief, EPCs were cultured in cell culture dishes not coated (vehicle group) or coated with biomaterial membranes (HA^*∗*^-Col I, HA-Col I^*∗*^, CS^*∗*^-Col I, or CS-Col I^*∗*^). When grown to 80% confluence, cells were washed with PBS and cultured in fresh medium for 48 h. Then the cell medium was collected and centrifuged at 300 g, 15 min, followed by 2000 g, 30 min, to remove cell debris. The cell-free culture medium was centrifuged at 20,000 g, 2 h to pellet MVs. The MVs were resuspended with 20 nm filtered (Whatman, Pittsburgh, PA) PBS. The number of cell particles was detected by the nanoparticle analyzer (NTA300, Malvern, Britain).

### 2.8. Analysis of miR-126

The level of miR-126 in EPC-MVs was determined by real-time PCR. Total miRs were extracted by using miRNeasy Mini kit (QIAGEN) following manufacturer's instructions. The miR-126 cDNAs were synthesized using Hairpin-it*™* miRNAs RT-PCR Quantization kit (GenePharma, Shanghai, China) based on manufactory instruments (25°C for 30 min, 42°C for 30 min, and 85°C for 5 min). Real-time PCR parameters were 95°C for 3 min; 40 cycles were performed at 95°C for 12 s and 60°C for 40 s [23]. PCR primers are as follows: 5-TATGGTTGTTCTCGACTCCTTCAC-3 and 5-TCGTCTGTCGTACCGTGAGTAAT-3 for miR-126; 5-CTC GCT TCG GCA GCA CA-3 and 5-AAC GCT TCA CGA AYY YGC GT-3 for U6. Quantitative real-time PCR was conducted on a real-time PCR system (Bio-Rad). Small nuclear RNA U6 (U6) was used as an internal control. Relative expression of miR-126 was calculated by using the 2^−ΔΔCT^ method [24].

### 2.9. Western Blot Analysis

Proteins from EPCs were extracted with lysis buffer. Protein lysates were electrophoresed through SDS-PAGE gel and transferred onto PVDF membranes. The membranes were blocked for 1 h and incubated with primary antibodies against caspase-3 (CST, USA) and *β*-actin (CST, USA) at 4°C overnight. After washing 3 times for 30 min with TBST, the immunoreactivity was visualized by ECL solution (GE Healthcare, USA).

### 2.10. Statistical Analysis

Data were all expressed as the mean ± SD. Multiple comparisons were performed by two-way ANOVA. Comparisons for two groups were performed by using Student's *t*-test (GraphPad Prism 5 software). *p* < 0.05 was considered significant.

## 3. Results

### 3.1. Multilayer HA/CS-Col I Membranes Similarly Inhibit EPC Apoptosis

**Figure 1:**
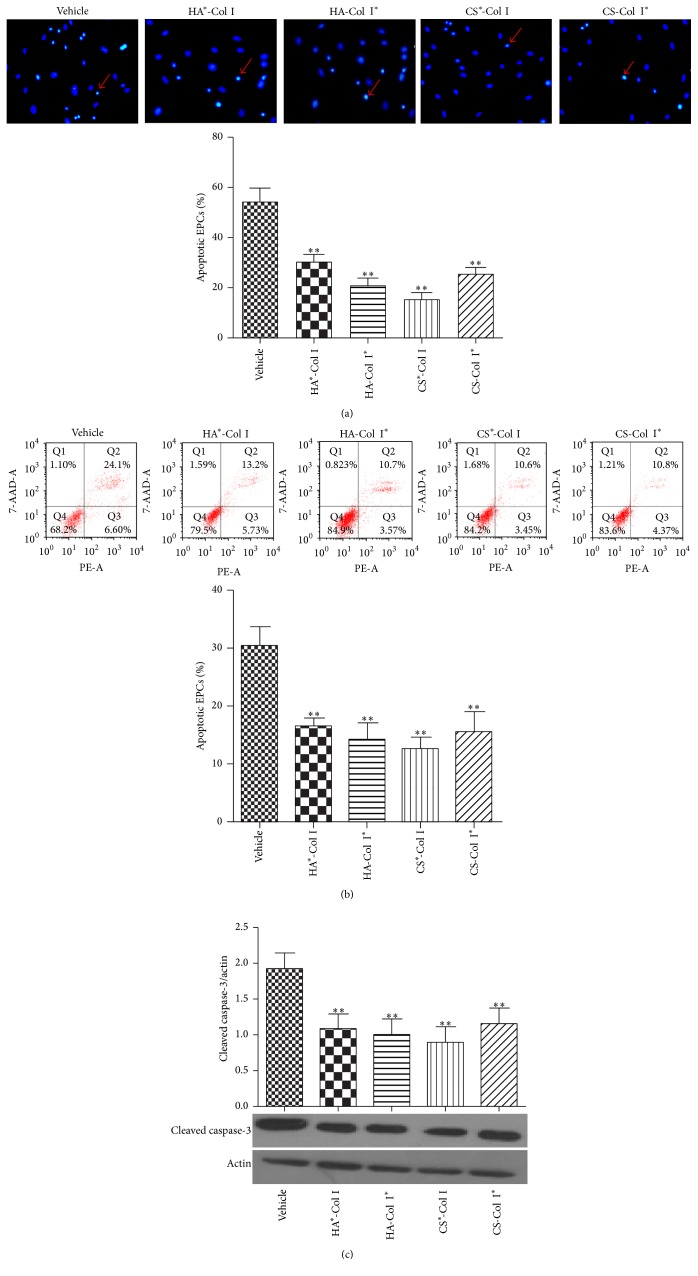
Effects of multilayer composite biomaterial membranes on EPC apoptosis. (a) EPC apoptosis determined by Hoechst 33258 staining (red arrows). (b) EPC apoptosis determined by Annexin V-PE/7-AAD staining and flow cytometric analysis. (c) Cleaved caspase-3 expression detected by western blot. ^*∗∗*^ *p* < 0.01 versus vehicle, *n* = 3/group.

Hoechst 33258 staining and Annexin V-PE/7-AAD analysis revealed that multilayer composite membranes significantly decreased serum-free culture induced EPC apoptosis (versus vehicle; Figures 1(a) and 1(b)). In addition, western blot data showed that the cleaved caspase-3 level, which was associated with induction of apoptosis, was inhibited by multilayer composite membranes (versus vehicle; Figure 1(c)). Interestingly, we did not find any significant differences of those measurements among the four multilayer composite membranes, indicating that all of them have antiapoptotic effects on EPCs.

### 3.2. Multilayer HA/CS-Col I Membranes Promote EPC Proliferation, with the Best Seen in *CS* ^*∗*^-Col I Membrane

**Figure 2:**
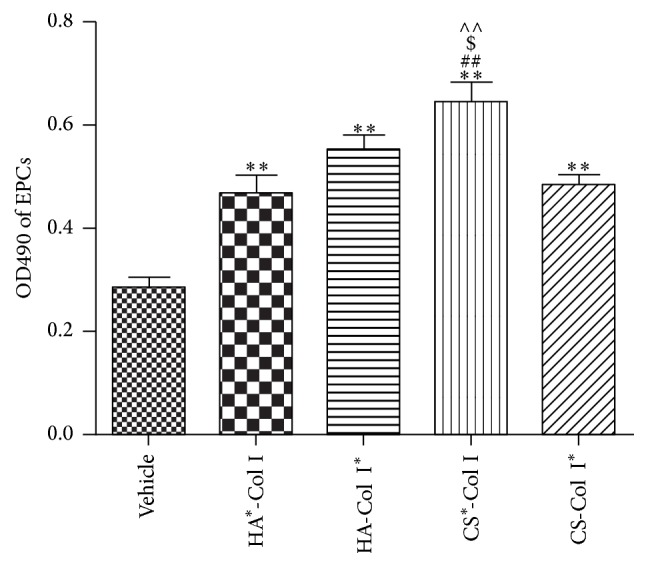
Effects of multilayer composite biomaterial membranes on the proliferation of EPCs. CS^*∗*^-Col I group showed the most significant effect on the EPC proliferation. ^*∗∗*^ *p* < 0.01 versus vehicle, ^##^ *p* < 0.01, ^$^ *p* < 0.05, and ^∧∧^ *p* < 0.01 versus HA^*∗*^-Col I, HA-Col I^*∗*^, or CS-Col I^*∗*^, *n* = 3/group.

MTT assay showed that all four multilayer composite membranes (HA^*∗*^-Col I, HA-Col I^*∗*^, CS^*∗*^-Col, and CS-Col I^*∗*^) markedly promoted EPC proliferation (1.6 ± 0.06-, 1.9 ± 0.13-, 2.3 ± 0.22-, and 1.7 ± 0.96-fold vehicle, resp.; Figure 2) when compared with untreated group. Meanwhile, we found that the CS^*∗*^-Col was most effective on increasing EPC proliferation. The OD490 value of CS^*∗*^-Col I treated EPCs was 1.39 ± 0.11-, 1.17 ± 0.07-, and 1.32 ± 0.08-fold HA^*∗*^-Col I, HA-Col I^*∗*^, and CS-Col I^*∗*^ treated EPCs, respectively (Figure 2).

### 3.3. Multilayer HA/CS-Col I Membranes Similarly Increase EPC Migration

**Figure 3:**
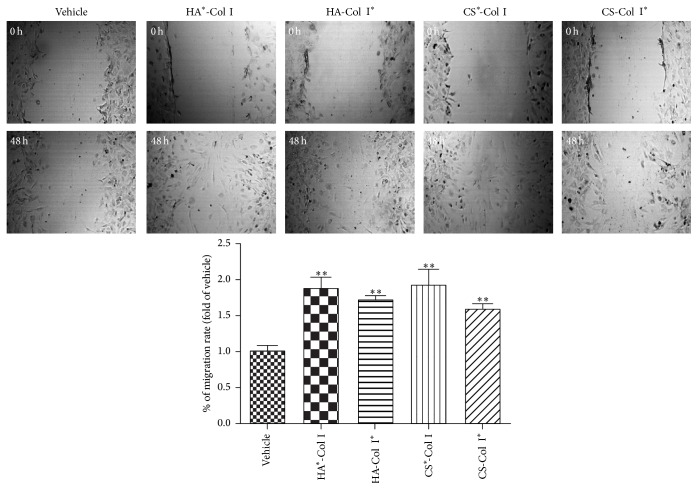
Effects of multilayer composite biomaterial membranes on the migration of EPCs. ^*∗∗*^ *p* < 0.01 versus vehicle, *n* = 3/group.

A scrape injury assay was carried out to assess the effect of multilayer composite membranes on EPC migration. The result revealed that all four multilayer composite membranes significantly increased the migration ability of EPCs, with the average migration area increased by 1.88 ± 0.15-, 1.72 ± 0.051-, 1.91 ± 0.23-, and 1.61 ± 0.062-fold vehicle, respectively, for HA^*∗*^-Col I, HA-Col I^*∗*^, CS^*∗*^-Col, and CS-Col I^*∗*^ treated EPCs (Figure 3). However, we did not find any difference among the four multilayer composite membranes.

### 3.4. Multilayer HA/CS-Col I Membranes Similarly Inhibit EPC Senescence

**Figure 4:**
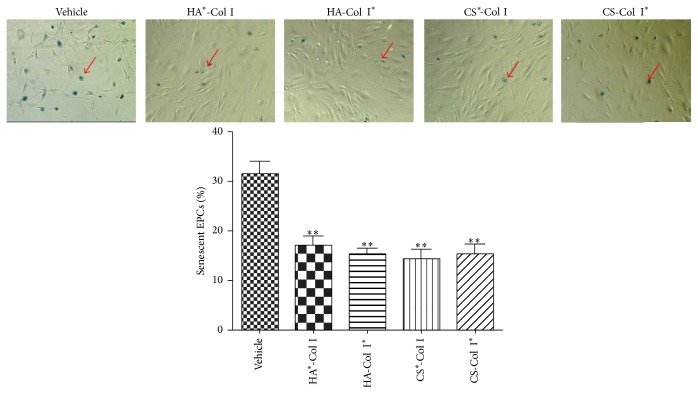
Effects of multilayer composite biomaterial membranes on senescent EPCs. Senescent EPC monitored by *β*-galactosidase staining (red arrows indicating apoptotic cells). ^*∗∗*^ *p* < 0.01 versus vehicle, *n* = 3/group.

To determine the cellular aging, *β*-galactosidase was used as a biochemical marker. In our experiments, EPCs showed 32.33 ± 3.05% senescence rate after long-term culture. As we expected, EPCs had reduced senescence rate when they grew on HA^*∗*^-Col I, HA-Col I^*∗*^, CS^*∗*^-Col, and CS-Col I^*∗*^ multilayer composite membranes (17.7 ± 3%, 15.5 ± 3%, 14.2 ± 1.51%, and 15.8 ± 3%, resp., Figure 4). In addition, we did not find any significant differences among the four groups.

### 3.5. Multilayer HA/CS-Col I Membranes Differently Increase EPC-MVs Releasing

**Figure 5:**
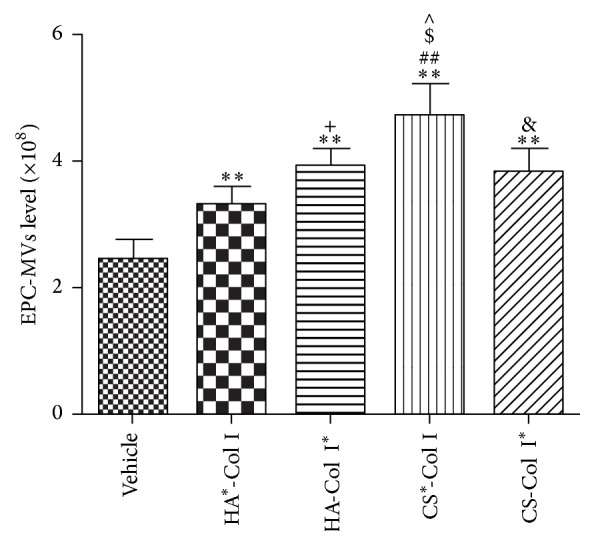
Effects of multilayer composite biomaterial membranes on the release of EPC-MVs. EPC-MVs level from CS^*∗*^-Col I group was higher than that from other biomaterial membrane groups. The level of EPC-MVs from HA-Col I^*∗*^ group was higher than that from HA^*∗*^-Col I group, and EPC-MVs level from CS-Col I^*∗*^ group was lower than that from CS^*∗*^-Col I group. ^*∗∗*^ *p* < 0.01 versus vehicle, ^##^ *p* < 0.01, ^$, ∧^ *p* < 0.05 versus HA^*∗*^-Col I, HA-Col I^*∗*^, or CS-Col I^*∗*^, ^+^ *p* < 0.05 versus HA^*∗*^-Col I, and ^&^ *p* < 0.05 versus CS^*∗*^-Col I, *n* = 3/group.

Our results showed that EPCs growing on HA^*∗*^-Col I, HA-Col I^*∗*^, CS^*∗*^-Col I, or CS-Col I^*∗*^ coated culture plate released more MVs (versus vehicle; Figure 5). We also found that the four types of membranes showed different effects on EPC-MV release. CS^*∗*^-Col I had the highest effect on stimulating EPC-MV release (versus HA^*∗*^-Col I, HA-Col I^*∗*^, or CS-Col I^*∗*^; Figure 5). The MVs release activity of EPCs was also different in other groups (HA-Col I^*∗*^, HA^*∗*^-Col I, and CS-Col I^*∗*^; Figure 5), suggesting that EPC-EM release is influenced by different biomaterials.

### 3.6. Multilayer HA/CS-Col I Membranes Differently Increase miR-126 Level in EPC-MVs

**Figure 6:**
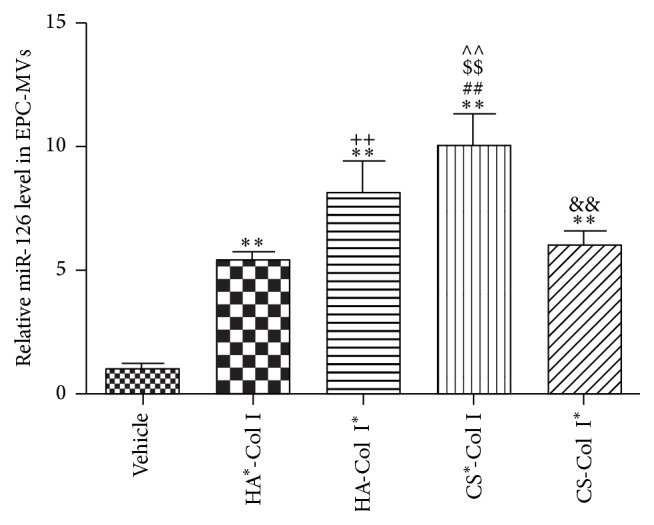
Effects of multilayer composite biomaterial membranes on the level of miR-126 in EPCs-MVs. miR-126 level in EPC-MVs from CS^*∗*^-Col I group was the highest. The miR-126 level in EPCs-MVs from HA-Col I^*∗*^ group was higher than that from HA^*∗*^-Col I group, and miR-126 in EPCs-MVs from CS-Col I^*∗*^ group was lower than that from CS^*∗*^-Col I group. ^*∗∗*^ *p* < 0.01 versus vehicle, ^##, $$, ∧∧^ *p* < 0.01 versus HA^*∗*^-Col I, HA-Col I^*∗*^, or CS-Col I^*∗*^, ^++^ *p* < 0.05 versus HA^*∗*^-Col I, and ^&&^ *p* < 0.05 versus CS^*∗*^-Col I, *n* = 3/group.

MV carried miRs could mediate the function of MVs. We found that the miR-126 in EPC-MVs was increased by the four different membranes (HA^*∗*^-Col I, HA-Col I^*∗*^, CS^*∗*^-Col I, or CS-Col I^*∗*^) after 24 h culture (Figure 6). We also found that effect of CS^*∗*^-Col was the highest (versus HA^*∗*^-Col I, HA-Col I^*∗*^, or CS-Col I^*∗*^; Figure 6). The miR-126 level in EPC-MVs from HA-Col I^*∗*^ was much higher than that from HA^*∗*^-Col I (versus HA^*∗*^-Col I; Figure 6), and that from CS-Col I^*∗*^ was lower than that from CS^*∗*^-Col I (versus CS-Col I^*∗*^; Figure 6).

## 4. Discussion

In this study, we studied the effects of four types of multilayer membranes (HA^*∗*^-Col I, HA-Col I^*∗*^, CS^*∗*^-Col I, and CS-Col I^*∗*^) on EPC functions and EPC-MV release. The major findings suggest that all the four types of membranes could promote EPC proliferation and migration and inhibit cell apoptosis and senility. The membranes could also promote the release of miR-126 rich EPC-MVs. Among them, the CS^*∗*^-Col I had the best effects on promoting EPC proliferation and miR-126 riched EPC-MV release.

Recent evidence suggests that surface modification with biomolecules like oligopeptides or glycans which can represent components of the ECM has been frequently applied to achieve an improved healing response of medical implants [25]. Biomaterial surfaces like glycosaminoglycans, such as HA and CS, have been found to enhance signal transduction into cells and promote osteoprogenitor cell spreading, proliferation, and differentiation [2, 3]. In this study, we found that biomaterial multilayer membranes composed of (HA/CS) and Col I promoted EPCs function. Moreover, the proliferation promoting effects were significantly enhanced when CS was applied on the top of CS^*∗*^-COI composed membrane, suggesting that CS might be more appropriate for EPCs.

EPCs are believed to play an important role in endothelial integrity, vascular homeostasis, and angiogenesis, which represents an important endogenous tissue repair and regenerative mechanism. Introduction or mobilization of EPCs can restore tissue vascularization after ischemic stroke and reestablish endothelial integrity [21, 26]. Various studies have attempted to enhance EPC functions under physiological and pathological conditions, so that they can be effectively used for therapy [27, 28]. Our group has reported that overexpression of CXCR4 and ACE2 could enhance the beneficial effect of EPCs-based therapy for ischemic stroke by promoting EPC proliferation and survival [29, 30]. The present study for the first time provides novel evidence regarding the effects of biomaterial membranes (HA/CS and Col I multilayers) on EPCs, which might be important when biomaterials and stem cells are applied in clinical therapy. The beneficial effects of biomaterial membranes for EPCs are likely through promoting cell proliferative activity, inhibiting apoptosis, and decreasing cell senescence. Several pathways may be involved in these effects. For example, Ras/ERK/VEGF and PI3K/Akt/eNOS signal pathways have been reported to be related with EPC proliferation, migration, and tube formation abilities [27, 28]. However, the precise underlying mechanisms need to be further investigated.

The therapeutic effect of stem cells is partly associated with their released MVs [19]. MVs not only carry the characteristics of mother cell source and are used as biomarkers, but also affect the functions of recipient cells through transferring the contents (proteins, miRNAs, and mRNAs) [31]. The numbers of circulating endothelial cells released MVs were demonstrated to increase in many vascular diseases including severe hypertension, acute coronary syndromes, and various forms of vasculitis [18, 32–34]. We have reported that EPC-MVs were negatively correlated with stroke severity and infarct volume in brain ischemic patient. Moreover, the increased level of EPC-MVs positively associated with vascular density in ischemic stroke [20]. MVs could be important underlying the mechanisms by which EPCs exert their functions. EPC-MVs have been reported to protect the kidney from ischemia-reperfusion injury and could enhance neoangiogenesis of human pancreatic islets [35, 36]. EPC-MVs could promote endothelial cell survival, proliferation, and tube formation ability, via the miR-126 in MVs [19], and play critical roles in angiogenesis and vascular integrity [37]. Circulating miR-126 which could exert an atheroprotective effect has been shown to be reduced in vascular diseases including stroke and coronary artery disease [38–40]. The miR-126 in EPC-MVs can be transferred to neighboring cells to modulate the activities of recipient cells. It was shown that miR-126 riched EPC-MVs ameliorated H/R induced HB-ECs apoptosis and dysfunction by activating PI3K/eNOS/NO pathway [20]. Jansen et al. reported that miR-126 was transported into human coronary artery endothelial cells (HCAECs) by endothelial cells released MVs (EMVs) and regulated HCAEC migration and proliferation [41]. These indicate that the level and carried contents of MVs are important factors closely related to the cell status and functions. EPC-MVs rich in miR-126 may exert beneficial effects on recipient cells. In the present study, we showed that the number and the miR-126 level of EPC-MVs from experimental multilayer membranes especially in CS^*∗*^-COI were all much more than those of the control group, indicating that the studied biomaterial membranes could promote EPC-MVs functions. In other words, they may help enhance EPCs therapeutic effects. The multilayer treatments improved EPC functions may be not only through regulating proliferation, migration, senescence, and apoptosis of EPCs, but also by releasing miR-126 riched MVs further promoting neighboring cell functions. However, this presumption needs to be verified by further investigations.

In addition, the number and the miR-126 level of EPC-MVs from membrane HA^*∗*^-Col I were strongly different from those of membrane HA-Col I^*∗*^. The number and the miR-126 level of EPC-MVs from CS^*∗*^-Col I were also significantly different from those of membrane CS-Col I^*∗*^. Therefore, we speculate that biomaterial which directly contacts with the EPCs plays a key role in regulating the release and miR-126 contents of EPC-MVs. This could help to choose the most effective biomaterial membranes for improving EPC functions when they are applied in clinical therapies.

## 5. Conclusion

In conclusion, biomaterials including HA^*∗*^-Col I, HA-Col I^*∗*^, CS^*∗*^-Col I, or CS-Col I^*∗*^ constructed multilayer membranes can modulate EPC functions and the number and miR-126 level of EPC-MVs. The beneficial effects of these biomaterial membranes are likely through promoting the proliferative, antiapoptotic, and antisenescence abilities of EPCs, as well as through enhancing EPC-MV release and miR-126 level. Therefore, combined use of biomaterials and stem cells may present a novel approach for promoting wound healing and tissue regeneration. However, further investigations in animal models and patients are required.
